# Simultaneous resurfacing of two fingers using a free medial plantar artery perforator flap: A case report

**DOI:** 10.1016/j.jpra.2025.06.021

**Published:** 2025-07-05

**Authors:** Chunyan Zhao, Toru Miyanaga, Masanobu Yamashita

**Affiliations:** Department of Plastic and Reconstructive Surgery, Kanazawa Medical University, 1-1 Daigaku, Uchinada, Ishikawa 9200293, Japan

**Keywords:** Medial plantar artery perforator flap, Fingertip reconstruction, Microsurgery, Traumatic amputation, Free flap transfer

## Abstract

This case report describes the reconstruction of two adjacent fingertip pulp defects using a single free medial plantar artery perforator flap. A 30-year-old man sustained traumatic amputations of the left index and middle finger pulps. A flap harvested from the medial plantar region was used to resurface both defects and was successfully anastomosed to the digital vessels of the index finger. Despite the need for a donor-site skin graft and secondary flap division, the procedure yielded excellent contour, mobility, and sensory recovery. The medial plantar artery perforator flap offers a reliable option with a good texture and color match, making it feasible even for general plastic surgeons without microsurgical subspecialization.

## Introduction

With the recent development of various perforator flaps, any skin deficit wound with exposure of bone, tendon, or other important structures can be covered without scarification of major muscles and/or vessels. Herein we report a case of reconstruction of the pulp of two fingers with one free medial plantar artery perforator flap.

## Case report

**Figure 1:**
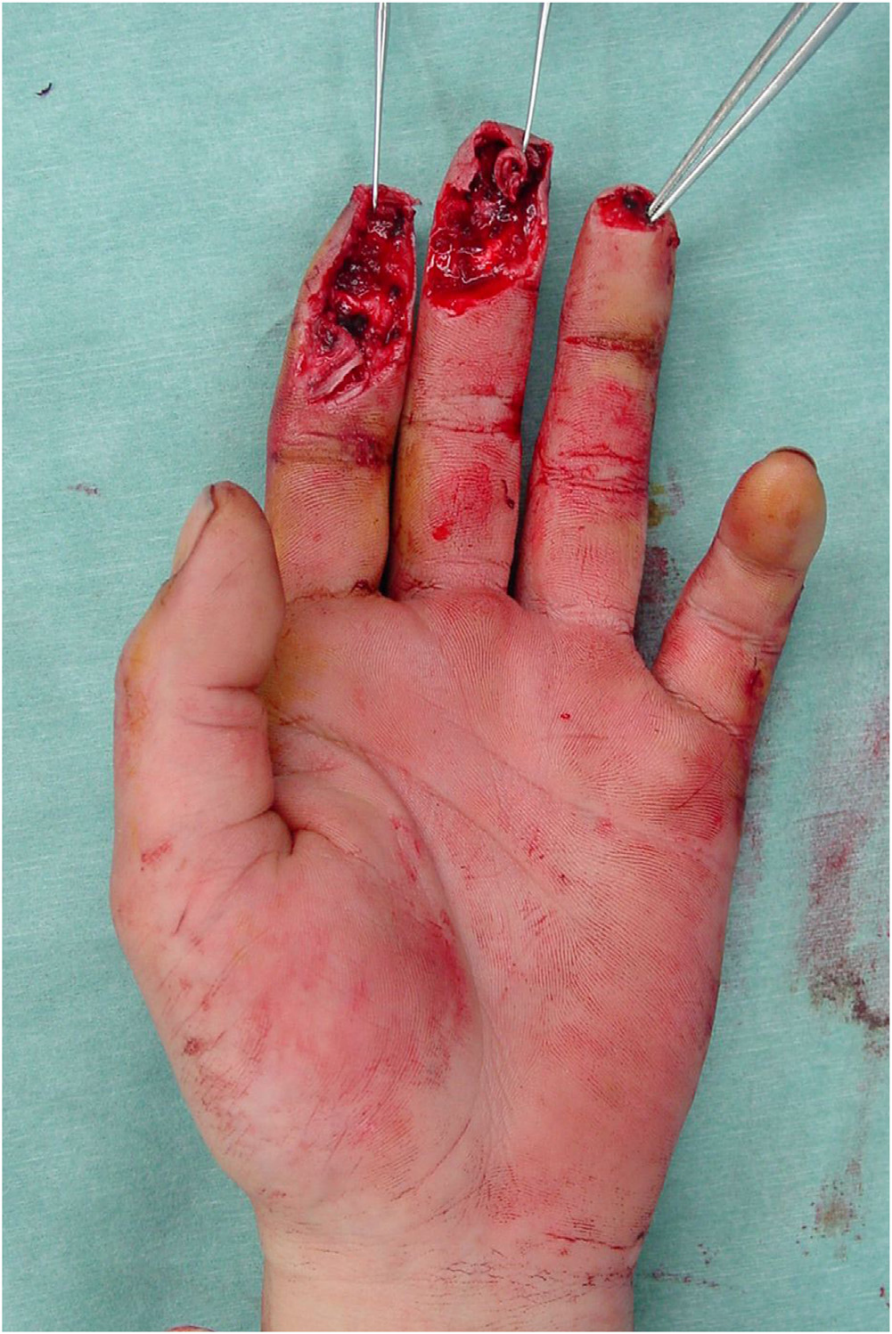
Intraoperative view of multiple soft-tissue defects on the volar aspect of the index and middle fingers, with bone and flexor tendon exposure.

**Figure 2:**
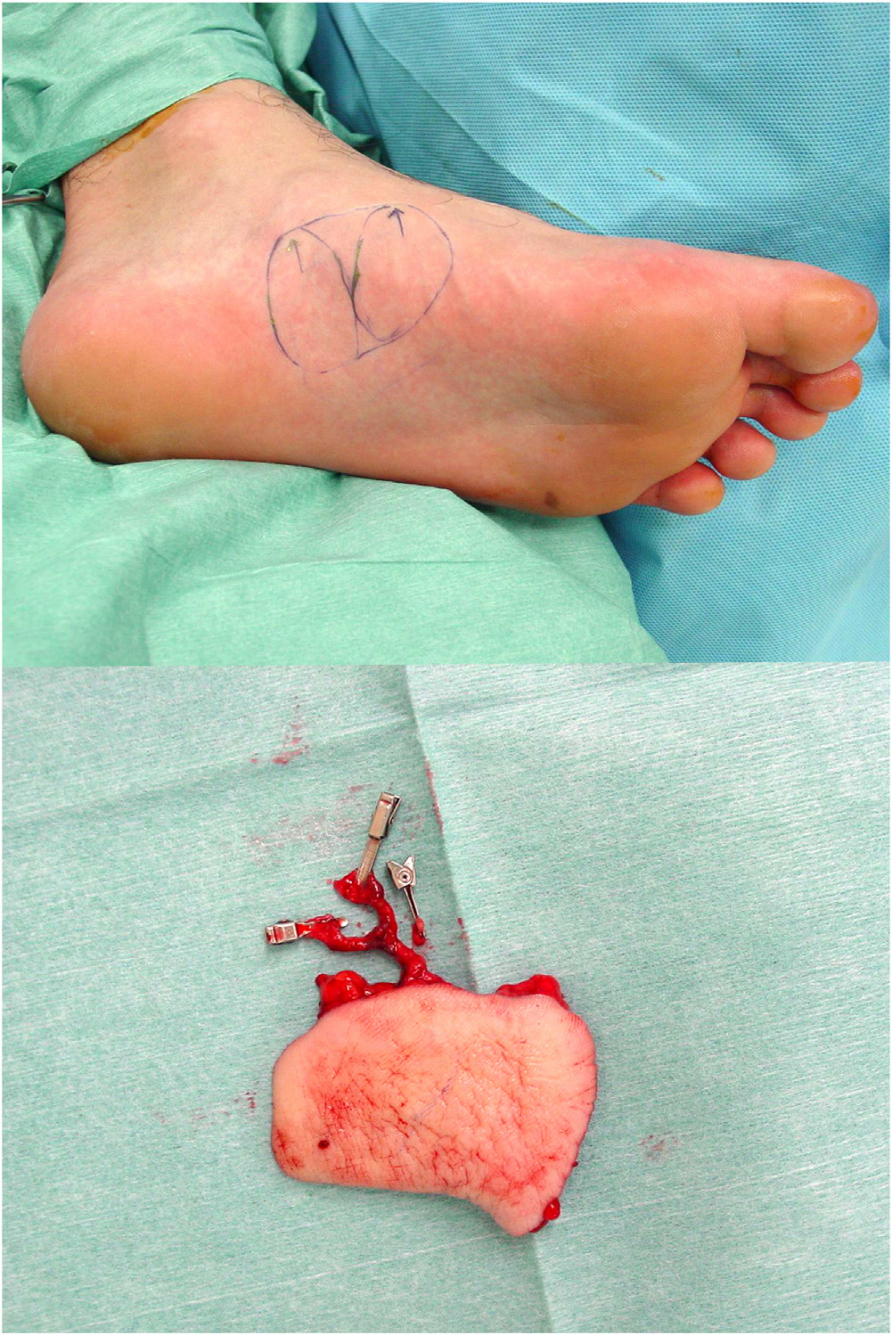
Free medial plantar artery perforator flap, including one perforator.

**Figure 3:**
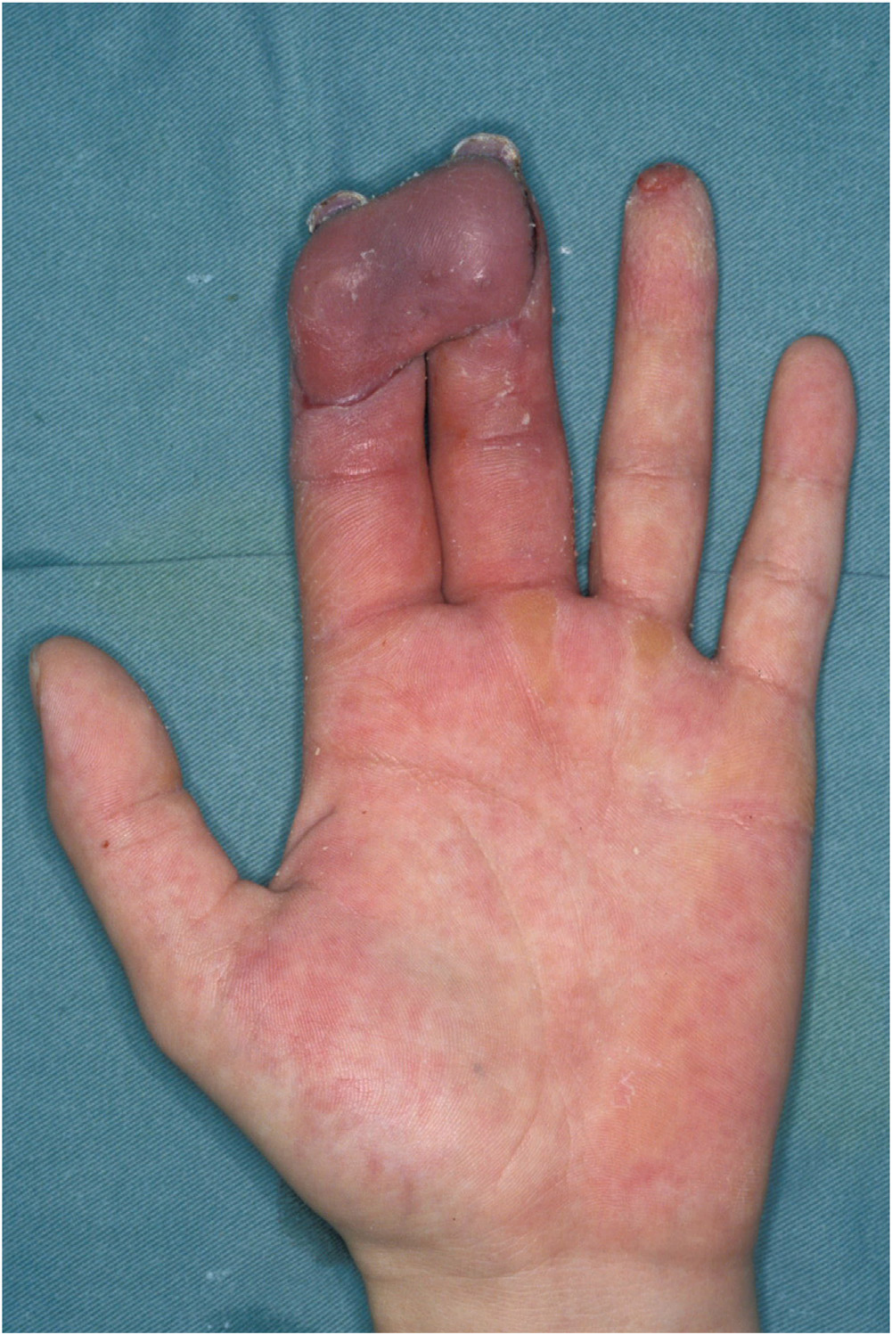
Postoperative view 3 weeks after the primary surgery.

**Figure 4:**
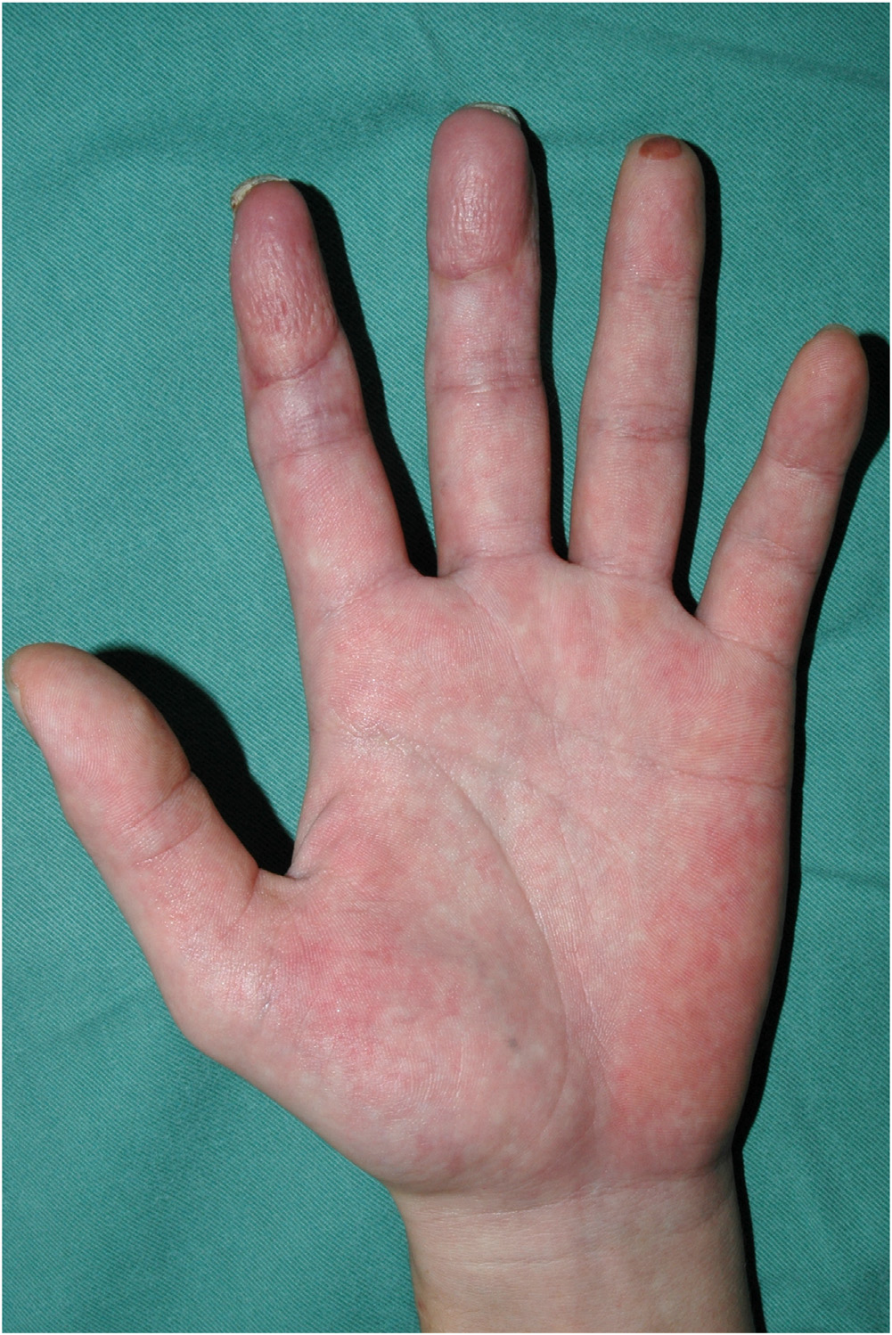
Postoperative view 8 months after the primary surgery.

A 30-year-old male presented to our institution with pulp amputations of his left index and middle fingers caused by an electric planer. Clinical examination showed defects in the skin and soft tissue, as well as exposure of the distal phalanges and flexor tendons. Preoperative x-ray examination revealed partial defects of the distal phalanx of both digits. No amputated tissue was retrieved. Surgery was performed as an urgent operation under general anesthesia on the day of injury. Kirschner wires were inserted through the tip of both fingers to stabilize the distal and proximal interphalangeal joints (Figure 1). A free medial plantar artery perforator flap was outlined on the patient’s left foot (Figure 2). The flap was elevated with one perforator (artery and concomitant vein). The defects of both fingers were resurfaced by the single flap and secured by 5–0 nylon sutures. Under microsurgical magnification, the medial plantar perforator artery was anastomosed to the ulnar common digital artery in the proximal portion of the index finger as a short pedicle using 10–0 nylon sutures, and the concomitant vein was anastomosed to the volar cutaneous vein of the index finger in an end-to-end fashion using 11–0 nylon sutures. The donor site was covered by full thickness skin grafting from the right groin region. One week later, range of motion exercises of the metacarpal phalangeal joints were begun. Three weeks after the first surgery, the flap was divided under local anesthesia to separate the two fingers without flap thinning (Figure 3). Eight months after the primary surgery, the contour and active range of motion of the fingers were almost normal (Figure 4), and the static two-point discrimination of both fingers was 5 mm.

## Discussion

Resurfacing of multiple finger amputations is complicated. Distant flaps, such as abdominal or groin flaps, are easy and safe to use, but they provide only fair color and texture match. Shoulder joint morbidity is also a potential problem with these flaps. Thenar flaps may be difficult to use for multiple finger defects because of the limited size of the donor site. If both tissue defect areas are relatively small, volar advancement flaps are an option. A reverse digital artery island flap is suitable for a moderate-size tissue defect of finger pulps. However, this flap has the disadvantage of requiring a skin graft to close the donor site on the injured finger, which may negatively influence postoperative rehabilitation and the final functional results.

Woo et al.^1^ reported the usefulness of venous free flaps for hand reconstruction, including 37 multiple digital defects reconstructed by a single flap. Their results were quite excellent. Li et al.^2^ described a technique for reconstructing multiple finger defects using a free multilobed posterior interosseous artery perforator flap. Satisfactory results were achieved in all 13 patients. The authors commented that the main disadvantage of this flap was the extensive dissection in the hand outside the zone of injury, to insert the flaps and gain access to recipient vessels when the defects were located distally and the skin paddles were small. Furthermore, skin grafting is required at the donor site for this flap, which can reduce the aesthetic satisfaction. Okada et al.^3^ reported the reconstruction of multiple adjacent fingers using combined medialis pedis and medial plantar fasciocutaneous flaps. In all three patients, the procedures were performed by a single, experienced hand and microvascular surgeon. These flaps provide excellent color and texture match but dissection of the vascular pedicle of these two combined flaps may be technically complicated. Ishikura et al.^4^ reported five cases of reconstruction using a medialis pedis flap and noted the anatomical variations of the flap’s vascular pedicle.

In the current case report, we resurfaced tissue defects on two adjacent fingers using one flap. Although this required a two-stage procedure, the flap was elevated with relative ease and did not require complicated vascular dissection. Indeed, the senior author was neither a hand surgeon nor microvascular surgeon. Reconstruction of finger pulp using a free medial plantar flap was initially reported by Inoue et al.^5^ in 1988. Use of a free medial plantar artery perforator flap was first reported by Koshima et al.^6^ in 2002 as a less invasive procedure for pulp in the palmer or plantar regions. The medial plantar artery perforator flap has several advantages. The main vascular branches, such as the posterior tibial and medial plantar arteries, can be preserved and deep dissection is not required. The flap provides excellent texture and color match for finger pulp. This flap also has two venous drainage systems: a concomitant vein and cutaneous vein. We chose the concomitant vein for venous drainage because sufficient venous return flow was observed from this vessel. As many authors have reported, satisfactory recovery of sensation is required for flaps covering the finger pulp to be considered successful. This was accomplished in the current case report.

Use of a medial plantar artery perforator flap is accompanied by the minor disadvantage of requiring a skin graft to close the medial instep donor region. Sole may be concerned about the exposure region, especially in some Asian countries where it is common custom to take off their shoes indoors. Although Koshima et al. indicated that it was difficult to detect and preserve the small perforator vessels and an experienced microvascular surgeon is needed, we believe that a medial plantar artery perforator flap can be the standard reconstructive procedure for general plastic surgeons when resurfacing multiple fingers.

## Funding

None.

## Patient consent

The patient provided written consent for the use of his images in the paper.

## Ethical approval

Not required.

## Conflict of interest

The authors have no conflicts of interest or funding to disclose.
